# Protein-protein interaction networks identify targets which rescue the MPP^+^ cellular model of Parkinson’s disease

**DOI:** 10.1038/srep17004

**Published:** 2015-11-26

**Authors:** Harriet Keane, Brent J. Ryan, Brendan Jackson, Alan Whitmore, Richard Wade-Martins

**Affiliations:** 1 Oxford Parkinson’s Disease Centre, Anatomy and Genetics, University of Oxford, OX1 3QX; 2 Department of Physiology, Anatomy and Genetics, University of Oxford, OX1 3QX; 3 e-Therapeutics plc, Long Hanborough, OX29 8LN; 4 Oxford Parkinson’s Disease Centre and Networks Cluster, Keble College, Oxford, OX1 3PG.

## Abstract

Neurodegenerative diseases are complex multifactorial disorders characterised by the interplay of many dysregulated physiological processes. As an exemplar, Parkinson’s disease (PD) involves multiple perturbed cellular functions, including mitochondrial dysfunction and autophagic dysregulation in preferentially-sensitive dopamine neurons, a selective pathophysiology recapitulated *in vitro* using the neurotoxin MPP^+^. Here we explore a network science approach for the selection of therapeutic protein targets in the cellular MPP^+^ model. We hypothesised that analysis of protein-protein interaction networks modelling MPP^+^ toxicity could identify proteins critical for mediating MPP^+^ toxicity. Analysis of protein-protein interaction networks constructed to model the interplay of mitochondrial dysfunction and autophagic dysregulation (key aspects of MPP^+^ toxicity) enabled us to identify four proteins predicted to be key for MPP^+^ toxicity (P62, GABARAP, GBRL1 and GBRL2). Combined, but not individual, knockdown of these proteins increased cellular susceptibility to MPP^+^ toxicity. Conversely, combined, but not individual, over-expression of the network targets provided rescue of MPP^+^ toxicity associated with the formation of autophagosome-like structures. We also found that modulation of two distinct proteins in the protein-protein interaction network was necessary and sufficient to mitigate neurotoxicity. Together, these findings validate our network science approach to multi-target identification in complex neurological diseases.

Biological phenotypes are underpinned by networks of molecular interactions that may themselves interact at mutiple levels. Network models built from molecular interaction data—e.g. protein-protein interactions—can capture important emergent properties of normal biological phenotypes such as robustness, whereas pathway representations cannot (see Young *et al.* (2012) for details)^1^,^2^. To destabilise this resilience, multiple, specific points within the data network must normally be perturbed^1^,^2^. It follows that modulation of a biological phenotype should require very specific, and generally multiple perturbations^2^,^3^,^4^. Analysis of network models of such phenotypic processes should allow such key intervention or control points in complex cellular systems, and particularly vulnerabilities in pathological states, to be identified. Using these principles, biological networks have been used to predict potential drug targets and to re-purpose existing drugs. Network science-based approaches are particularly suited to the analysis of multifactorial diseases, enabling more accurate representation of the complexity of the interplay between multiple different cellular processes^2^,^4^. It has therefore been suggested that network-based approaches might be particularly suited to help develop novel therapeutics for complex neurodegenerative diseases^5^.

Cell death in Parkinson’s disease (PD) is not fully understood and there are currently no treatments capable of curing or slowing disease progression. A number of key pathological cellular processes have been identified in PD including oxidative stress and dysregulation of autophagy^6^,^7^. Additionally, mitochondrial dysfunction is known to be central to PD pathology and both somatic and inherited mitochondrial mutations have been shown to contribute to the disease^8^. Indeed, the exquisite sensitivity of A9 dopaminergic neurons of the substantia nigra has been linked to their uniquely high energy demands and it is hypothesised that a systemic mitochondrial dysfunction is most likely to present as deleterious in this vulnerable population^9^. It is the interplay between multiple hits, such as this bioenergetic burden, in combination with increased cellular stresses, which has been hypothesised to lead to neurodegeneration in PD^7^,^8^, thus making the disease an exemplar for systems biology approaches.

Mitochondrial complex I inhibition is a feature of both familial and sporadic Parkinson’s disease, with complex I inhibitors also inducing PD-like disease in humans and animal models^10^,^11^. The neurotoxin MPP^+^, the active metabolite of MPTP, is a potent complex I inhibitor inducing ATP depletion and apoptotic cell death and is widely used to acutely model mitochondrial complex I dysfunction-induced cell death in cell cultures^12^,^13^,^14^. Impairments in autophagy and the accumulation of aggregrated proteins into Lewy Bodies are key features of post-mortem PD brain. Aggregation of α-synuclein, the major component of Lewy bodies is a key component of disease aetiology both as a substrate and inhibitor of autophagic processes^15^,^16^. MPP^+^ treatment has also been demonstrated to alter autophagic flux *in vitro*, although the direction of change appears to be dependent on cell type and MPP^+^ concentration^17^,^18^, with recent reports of inhibition of autophagy both potentiating and/or rescuing MPP^+^ toxicity^19^,^20^. Autophagy is particularly important in long lived cells such as neurons^21^, and knockout of essential autophagy proteins has been shown to lead to neurodegeneration even in the absence of disease related proteins^22^. In addition, stimulation of autophagy by rapamycin or other compounds has been demonstrated to protect cells from MPTP toxicity^23^,^24^. The processes of mitochondrial dysfunction and autophagy are intimately linked with damaged mitochondria broken down through the process of mitophagy^25^,^26^.

Here, we have tested the hypothesis that analysis of protein-protein interaction networks can be used to identify protein target sets, which when modulated in concert, yield therapeutic benefit in cellular models of pathology. Sub-networks representing autophagy and mitochondrial dysfunction in an MPP^+^ model were created and their union formed. Four proteins (P62, GABARAP, GBRL1 and GBRL2) with a high betweenness in the union network were identified. Our network analysis predicted these proteins to be important for the cross talk between mitochondrial dysfunction, autophagy and MPP^+^ neurotoxicity in the pathological state. Overexpression of the network target proteins in combination, but not individually, achieved a rescue of the MPP^+^ neurotoxin cellular model of PD, suggesting significant functional (and non-linear) complexity in the pathological networks that is tractable though a network science approach.

## Results

### Analysis of protein-protein interaction networks identifies key mediators of neurotoxicity

Publically available protein-protein interaction data obtained from the iRef Index database^27^ were curated to remove all non-human species and predicted interactions, providing a database of 15,298 proteins and 90,628 interactions. This dataset was sampled to create smaller networks that were enriched in MPP^+^ relevant processes. To model the roles of autophagy and mitochondrial dysfunction in MPP^+^ toxicity a seed list was created for each process (Supplementary Tables 1 and 2).

**Figure 1:**
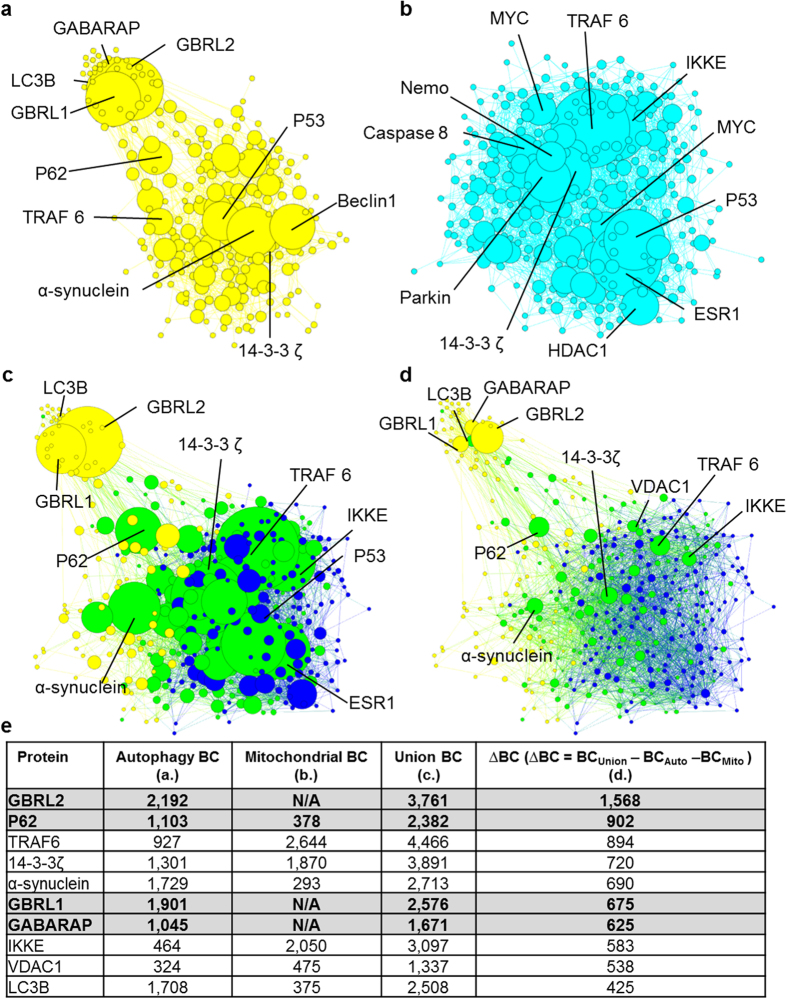
Protein-protein interaction networks identify candidate targets that modulate MPP^+^ toxicity. (**a**,**b**) Protein-protein interaction networks representing mitochondrial dysfunction and the dysregulation of autophagy following MPP^+^ exposure were created. Seedlists of proteins with validated roles in either (**a**) autophagy (yellow) or (**b**) mitochondrial dysfunction (cyan/blue) were used to sample iRef Index. Nodes are sized according to betweenness centrality (BC) with the ten nodes with the highest value of BC labelled. (**c**,**d**) A union network representing cross talk between mitochondrial dysfunction and autophagy was formed. Nodes which are common to both networks are coloured green and size represents overall BC in the union network (**c**), or ΔBC (BC_Union_ − BC_Auto_ − BC_Mito_) (**d**) to represent the importance of each node in linking these two processes together. (**e**) Targets for *in vitro* validation were selected. BC values for the 10 nodes with highest ΔBC as represented in each of the four networks – highlighted proteins were investigated experimentally and N/A indicates that the node does not appear in the sub-network and therefore has no BC.

Seed lists were used to sample from the experimental human-human protein-protein interaction dataset created above using a shortest path approach^28^ to create two sub-networks (Fig. 1a,b). The union of these two sub-networks was formed to represent the interaction between these processes at a cellular level. Betweenness centrality (BC; a measure of the number of shortest paths across the network that include any given node) was calculated for each node in the two sub-networks and the union network (Fig. 1a–c). The ∆BC was defined as the difference between the BC in the union network and the sum of this value in the two sub-networks 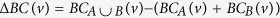 (Fig. 1d). ∆BC is a measure of the importance of each node in connecting autophagy and mitochondrial dysfunction in the MPP^+^ system and we hypothesised that manipulating nodes with high ∆BC would modulate MPP^+^ toxicity.

Using this analysis, gamma-aminobutyric acid receptor-associated protein like protein 2 (GBRL2) and P62 were identified as the nodes with the highest ΔBC values. (Figure 1e). GBRL2 is a member of the GABARAP subfamily, mammalian orthologs of the yeast protein ATG8, which have been shown to be important for autophagosomal maturation. Interestingly, GBRL1 and GABARAP, proteins with high sequence homology to GBRL2, were also found to exhibit high-ranking ΔBC values (6^th^ and 7^th^ respectively). Given the homology and functional redundancy observed in this subfamily^29^,^30^, GBRL1 and GABARAP were included in the targets for *in vitro* validation. P62 also has a critical role in autophagy and acts as an adaptor allowing ubiquitinated cargo to be targeted for degradation^31^. Western blot confirmed that two of the target proteins, P62 and GABARAP, were expressed at the protein level in BE(2)-M17 cells (Supplementary Fig. 1a–c) – a previously-established dopaminergic cellular model of PD^14^. However, none of the targets demonstrated differential mRNA or protein expression following MPP^+^ treatment (Supplementary Fig. 1d–g).

Interestingly, α-synuclein was also identified as critical for the cross talk between autophagy and mitochondrial dysfunction (ranked 5^th^). We and others have previously demonstrated that knockdown of α-synuclein both *in vitro* and *in vivo* is capable of rescuing multiple cell types from MPP^+^ toxicity^13^,^14^,^32^,^33^ and this potential network bridging role in the pathological state provides some insight into how this rescue may be explained in network terms.

### Combined knockdown of proteins identified through network analysis sensitises cells to MPP^+^ cytotoxicity

Consistent with our previous observations, MPP^+^ caused cell death in a time and dose-dependent manner. This toxicity occurs via ATP-depletion, loss of mitochondrial membrane potential ultimately leading to cell death (Supplementary Fig. 2). Additonally, short-term MPP^+^ treatment (5 h; prior to significant MPP^+^-induced cell death) also results in lysosomal deacidification, as assessed by uptake of the lysosomal pH-sensitive dye neutral red in a smilar manner to that observed by the vacuolar-type ATPase proton pump inhibitor bafilomycin (Supplementary Fig. 2d).

**Figure 2:**
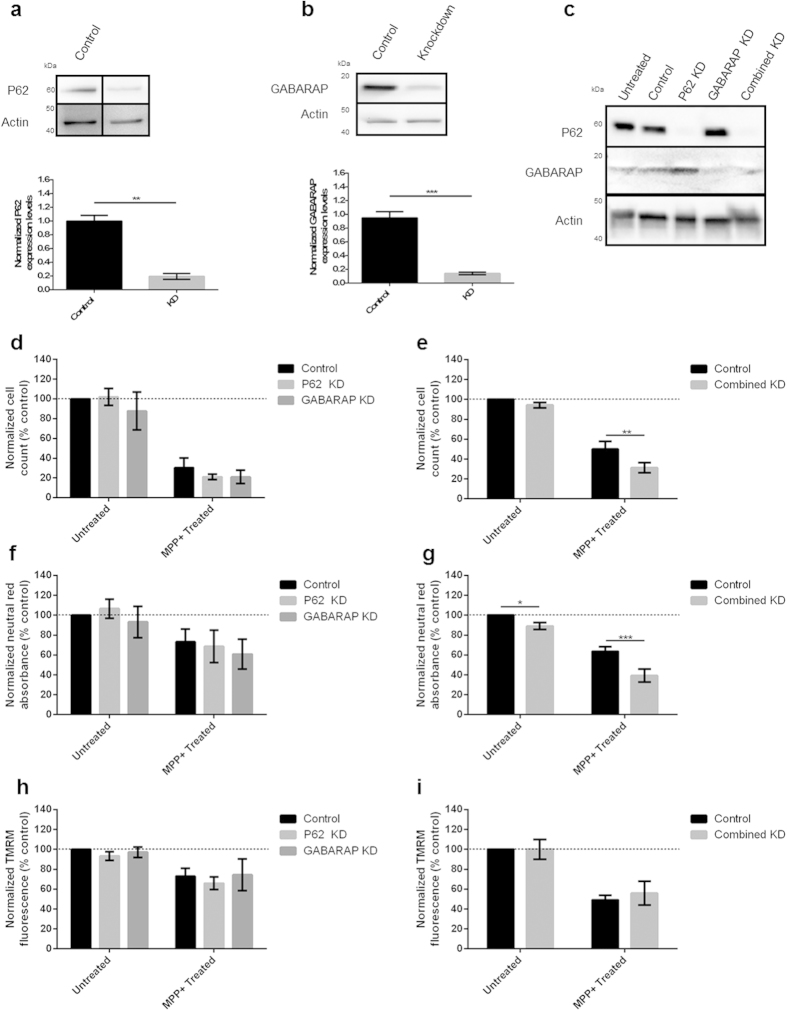
siRNA knockdown of key autophagy proteins sensitises cells to MPP^+^ induced cell death. (**a**–**c**) Efficient RNAi-mediated knockdown of P62 and GABARAP in BE(2)M17 cells. Cells transfected with appropriate targeting or scrambled control siRNAs (50 nM) were harvested 72 h after transfection. Protein levels were measured by western blot and band density was normalised relative to actin. Representative images are shown (n = 3). (**d**–**i**) RNAi-mediated knockdown of P62 and GABARAP in BE(2)M17 cells exacerbates sensitivity to MPP^+^. Cells were transfected with appropriate targeting or scrambled control siRNAs (50 nM), treated with MPP^+^ (100 μM) and assayed. Combined KD indicates simultaneous P62 and GABARAP knockdown. (**d**,**e**) Cell death was assessed by counting the number of morphologically normal cells in bright field images at 48 h post-MPP^+^ treatment. (**f**,**g**) Cell viability was assessed by neutral red uptake at 36 h post-MPP^+^ treatment. (**h**,**i)** Mitochondrial membrane polarisation was measured using TMRM fluorescence 24 h post-MPP^+^ treatment. Bars represent mean values normalised to untreated control cells ± SEM (n = 3), data were analysed using a two way ANOVA with Sidak multiple comparison test *P ≤ 0.05, **P ≤ 0.01, ***P ≤ 0.001.

In order to model node deletion, P62 and GABARAP were knocked-down (KD) using siRNA by 81 and 86% respectively, as assessed by western blot (Fig. 2a,b). Simultaneous knockdown of P62 and GABARAP did not reduce efficiency of knockdown (Fig. 2c). KD of GBRL1 and GBRL2 was not attempted due to the low basal expression levels (Supplementary Fig. 3c). KD of either P62 or GABARAP individually had no effect on cell viability, as assessed by cell counts, the lysosomal pH-sensitive dye neutral red or mitochondrial membrane potential as assessed by TMRM fluorescence (Fig. 2d,f,h). However, a combined knockdown of both P62 and GABARAP led to increased cellular vulnerability in the presence of MPP^+^ as demonstrated by reduced cell counts and neutral red uptake (Fig. 2e,g). No change in mitochondrial membrane potential was observed following the combined KD and MPP^+^ treatment (Fig. 2i), suggesting that the effects of protein modulation lie downstream of mitochondrial toxicity.

### Combined overexpression of multiple proteins identified through network analysis rescues MPP^+^ induced cell death

**Figure 3:**
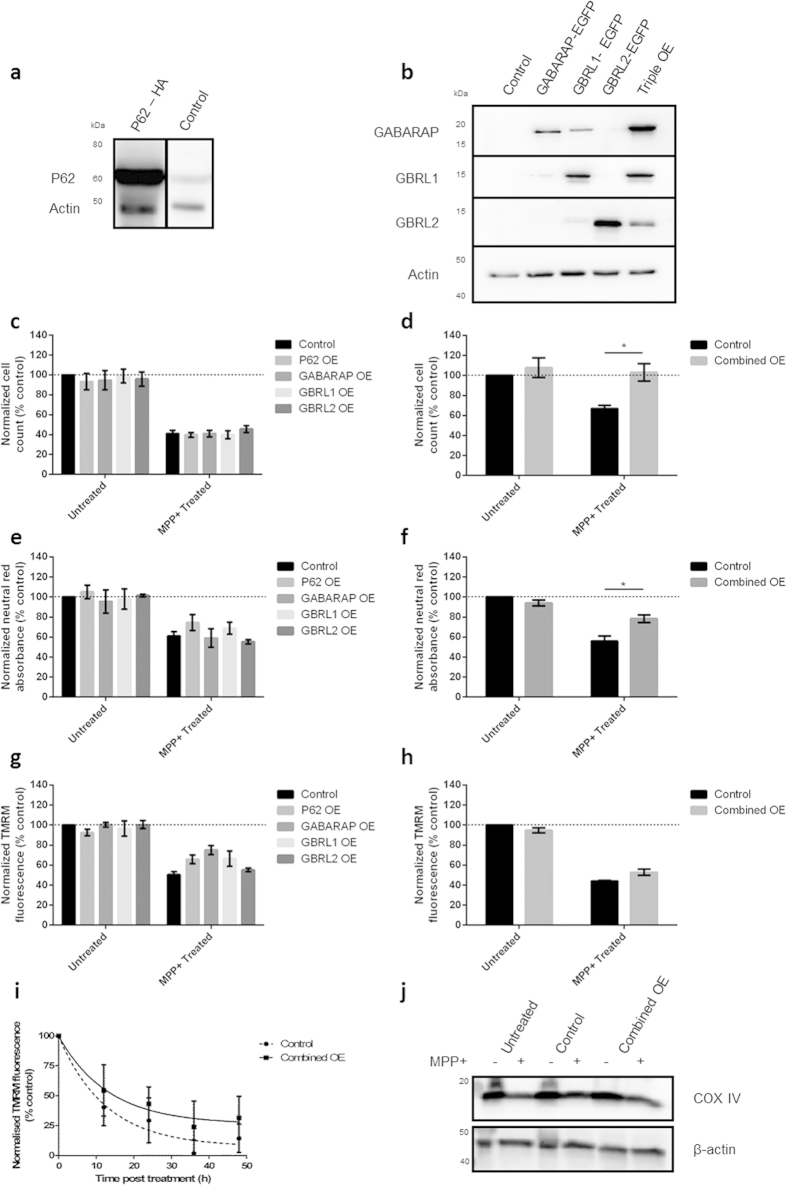
Overexpression of key autophagy proteins protects cells against MPP^+^ induced cell death. (**a**,**b**) Overexpression of P62, GABARAP, GBRL1 and GBRL2 in BE(2)-M17 cells. Cells transfected with appropriate plasmids were harvested 72 h after transfection. Protein levels were measured by western blot and band density was normalised relative to actin. (**c**–**h**) Simultaneous overexpression of P62, GABARAP, GBRL1 and GBRL2 rescues MPP^+^ toxicity. Cells were transfected with expression constructs, treated with MPP^+^ (100 μM) and assayed. 4OE indicates simultaneous overexpression of P62, GABARAP GBRL1 and GBRL2. (**c**,**d**) Cell death was assessed by counting the number of morphologically normal cells in bright field images 48 h after MPP^+^ treatment. (**e**,**f)** Cell viability was assessed by neutral red uptake 36 h after MPP^+^ treatment. (**g**–**i**) Mitochondrial membrane polarisation was measured using TMRM fluorescence (**g,h**) 24 h after MPP^+^ treatment or (**i**) after 12, 24, 36 or 48 h MPP^+^ treatment. (**j**) Representative western blot of the mitochondrial protein COX IV 48 h after MPP^+^ treatment in the presence/absence of combined protein overexpression (n = 3). Bars represent mean values normalised to untreated control cells ± SEM (n = 3), data were analysed using a two way ANOVA with Sidak multiple comparison test * = P ≤ 0.05.

Given the deleterious effects of combined KD of the network targets, the effect of target protein overexpression was investigated. Overexpression of each of the four target proteins was achieved using cDNA expression constructs, as assessed by western blot (Fig. 3a,b). As previously reported^34^, there is some cross-reactivity between GABARAP and GBRL1 antibodies. Additionally, in the case of the EGFP labelled GABARAP subfamily proteins, protein expression was assessed by fluorescence microscopy (Supplementary Fig. 3a). Overexpression of multiple constructs did not increase the number of EGFP-positive transfected cells and similar protein levels were achieved in the single and triple transfections, confirming the feasibility of this approach (Supplementary Fig. 3b).

No change in cell viability or MPP^+^ toxicity was observed following overexpression of individual target proteins, as measured by cell counts, neutral red uptake or TMRM fluorescence (Fig. 3a,c,e). However, simultaneous overexpression of the four network targets resulted in rescue of MPP^+^-induced cell death as observed by cell counts and neutral red absorbance (Fig. 3d,f) confirming that multiple points of intervention are required to rescue the cellular model. This protection was independent of changes in TMRM fluorescence, indicating the mechanism of rescue lies downstream of mitochondrial toxicity (Fig. 3h). Furthermore, measurement of TMRM at 12h intervals demonstrated that no statistically significant rescue of TMRM was observed at any of the time points tested, suggesting that overexpression of multiple network targets does not rescue cells by retarding mitochondrial depolarisation (Fig. 3i). Consistent with these obsevations, combined overexpression of network targets does not rescue the reduction of the mitochondrial protein COX IV following MPP^+^ treatment (Fig. 3j).

### Network target overexpression results in increased autophagosome formation

**Figure 4:**
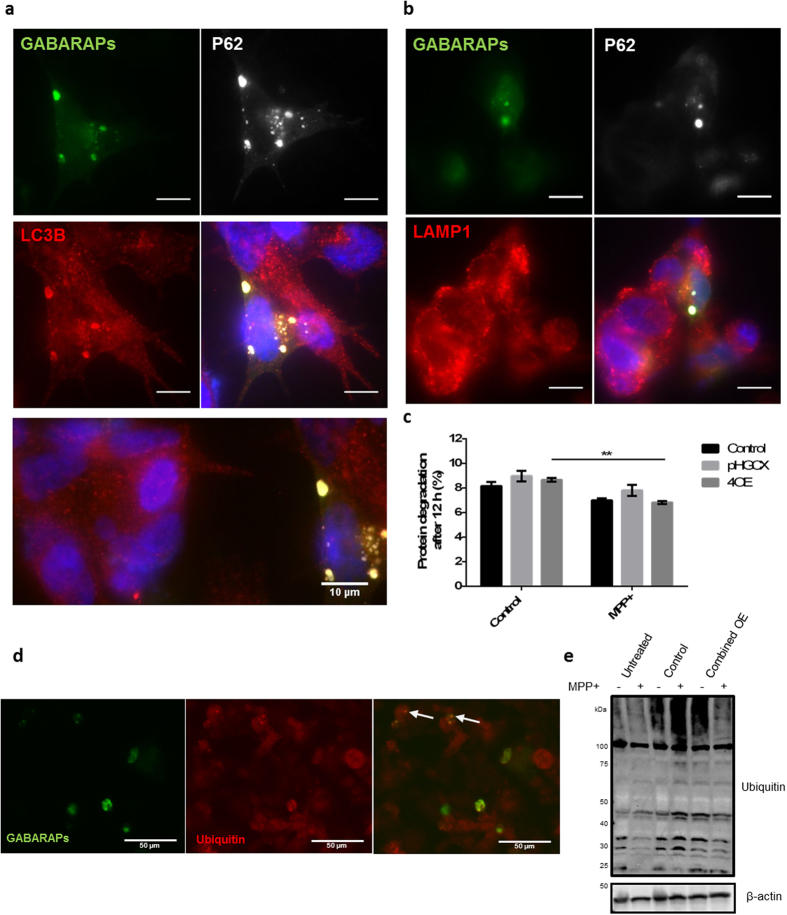
Cell protection is associated the formation of autophagic bodies, but not by increased protein degradation. (**a**,**b**) Prominent autophagosomes formed in transfected cells but did not fuse with lysosomes. Cells transfected with the combined overexpression set (P62, GABARAP, GBRL1 and GBRL2) were fixed and stained for P62, LAMP1 and LC3B 48 h after transfection. Expression of GABARAPs was shown by EGFP fluorescence. Untransfected cells are shown to the left of the bottom image in (**a**). (60 × magnification, scale bar represents 10 μm). The combined overexpression did not alter (**c**) protein degradation or (**e**) protein ubiquitination. (**c**) 14 C labelled cells were transfected with the combined plasmid set or control (pHGCX) plasmid and the rate of protein breakdown measured in both presence and absence of MPP^+^. Bars and data points represent mean values normalised to untreated control cells ± SEM (n = 3), data were analysed two way ANOVA with Sidak’s multiple comparison test to control, **P ≤ 0.01. (**d**) Representative image of cells overexpressing network targets stained for ubiquitin, arrows indicate GABARAP (green)/ubiquitin (red) colocalisation. Cells were imaged at 20 × magnification 48 h after transfection. (**e**) Representative western blot of protein ubiquitination after MPP^+^ treatment cells overexpressing network targets. Cells were harvested 36 h post-MPP^+^ treatment and protein ubiquitination was assessed by western blot (n = 3).

Given the observation that overexpression of network targets rescued cells from MPP^+^ toxicity, independent of rescue of mitochondrial function, we further investigated the mechanism of this protection. The combined overexpression of target proteins resulted in the formation of prominent LC3B-positive autophagosomes, which were not present in untransfected cells, consistent with increased autophagic capacity in cells overexpressing target proteins (Fig. 4a). However, these autophagosomes did not colocalise with the lysosomal membrane protein LAMP1 (Fig. 4b). The lack of colocalisation between P62/GABARAP positive structures with lysosomes may indicate a failure of lysosomal fusion, which may be a result of the decreased lysosomal pH and ATP depletion observed after MPP^+^ treatment (Suplementary Fig. 2d). Furthermore, overexpression of network targets did not atennuate the MPP^+^-dependent decrease in the breakdown of long-lived proteins, as measured by radio-labelled valine release, despite BE(2)-M17 cells being amenable to increased rapamycin-mediated protein degradation (Fig. 4c and Suplementary Fig. 4c). Consistent with the observed increase in P62/GABARAPs/LC3 positive structures and the failure of autophagic protein breakdown after MPP^+^ treatment, we observed that MPP^+^ treatment resulted in increased LC3 lipidation (Supplementary Fig. 4a,b).

It was also observed that these P62/GABARAPs/LC3-positive structures partially colocalise with accumulations of ubiquitinated cargo (Fig. 4d). In parallel, no significant changes in protein ubiquitination were observed when network targets were overexpressed in concert (Fig. 4e). Together, these observations indicate that combined over-expression of the selected proteins leads to increased capacity to form autophagosome-like structures improving the efficiency of the initial stages of autophagy or acting to sequester autophagic cargo, consistent with the observed protection from MPP^+^ toxicity.

### Network targets demonstrate functional redundancy in neuro-protective effe**cts**

**Figure 5:**
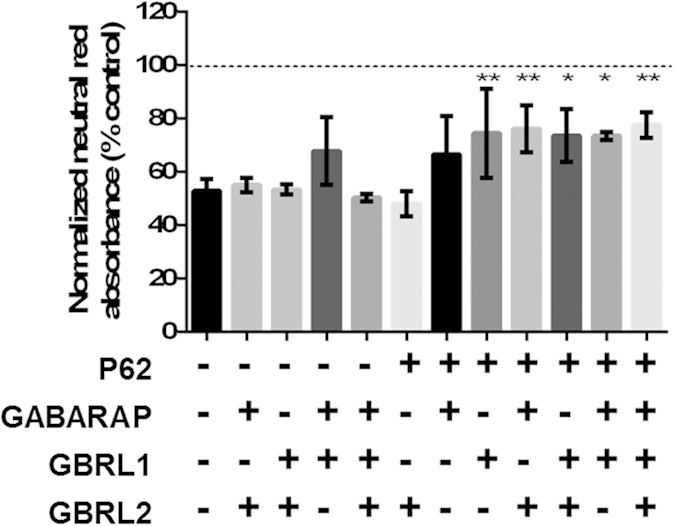
A minimal overexpression set contains P62 and members of the GABARAP subfamily. All pairwise and triple combinations of the overexpression set were tested for rescue of MPP^+^ toxicity. Cells were transfected with appropriate expression constructs, treated with MPP^+^ (100 μM) and neutral red uptake measured 36 h post-MPP^+^ treatment. Bars represent mean ± SEM (n = 3), one way ANOVA with Dunnett multiple comparison test to MPP^+^ control, *P ≤ 0.05, **P ≤ 0.01.

Finally, we investigated potential redundancy in our selected network targets to understand if overexpression of all target proteins is needed for the observed neuro-protective effect. Each possible pairwise and triple combination of network targets was overexpressed and vulnerability to MPP^+^ assessed. We determined P62 was essential for cellular rescue and that there was considerable redundancy in the GABARAP subfamily proteins, with any combination of two GABARAP subfamily members, or GBRL1 alone, sufficient for neuroprotection (Fig. 5). It has previously been demonstrated that a combined knockdown of all GABARAP subfamily members is required to elicit an autophagic phenotype^35^. These data demonstrate that interventions are required at multiple distinct positions in the protein-protein interaction network to achieve a phenotypic outcome, suggesting that, in common with networks underpinning normal function, this pathological network is resilient, exhibiting considerable redundancy, and that non-trivial techniques are required to identify vulnerabilities.

## Discussion

The identification of novel therapeutic targets in complex diseases, such as PD, is a major challenge in developing effective therapies for such disorders. Here we demonstrate that network analysis can identify proteins which, when modulated in concert, alter neurotoxicity in an *in vitro* model of PD. These observations demonstrate the potential of a workflow encapsulating creation of *in silico* pathological networks, through target identification to phenotypic benefit in a model of neurological disease.

We have modelled mitochondrial dysfunction and autophagy, two key processes in both PD and MPP^+^ toxicity, using a shortest-path sampling approach to create a protein-protein interaction network enriched in relevant MPP^+^/PD proteins. The union of the sub-networks reflecting mitochondrial dysfunction and autophagy was formed and the nodes vital to the transmission of information were identified using an algorithm based on BC. Four target nodes/proteins (P62, GABARAP, GBRL1 and GBRL2) were identified and represent both cargo-targeting (P62) and autophagosome formation (GABARAP subfamily), two processes involved in macroautophagy/mitophagy.

siRNA-mediated knockdown of target proteins only potentiated MPP^+^ toxicity when targets were knocked down in concert, indicating a requirement for multiple interventions to elicit phenotypic effects. Both theoretical network studies and experiments in yeast have indicated that normal networks are generally robust to single deletions and that, to overcome this, deletions must be both multiple and targeted unless the network is in a very unusual state^1^,^2^. Experimental results indicate that although biological systems are robust to single deletions under optimal conditions, they may be vulnerable when these are combined with experimental stress or pre-existing deletions—the concept of synthetic lethality^36^. For example, in the case of MPP^+^ toxicity, the resultant pathological network might have atypical characteristics that render it more vulnerable (than the individual networks from which it is derived) to attack by small numbers of particularly pleiotropic interventions (such as single knockdown of α-synuclein). Although complex, the MPP^+^ driven network is not *a priori* a result of evolutionary selection and it does not itself underpin a robust and essential biological function. In contrast, knockdown of α-synuclein in normal cells does not impact their function suggesting highly evolved robustness and redundancy in normal α-synuclein function e.g. presynaptic vesicle trafficking.

Conversely, overexpression of the four proteins identified by network analysis resulted in significant protection against MPP^+^-induced cell death. However, mirroring siRNA knockdown, upregulation of individual proteins in isolation was insufficient to affect this cell loss significantly. We demonstrated that upregulation of at least two of the four network targets was required to elicit the protective effect, provided one of the targets was P62, confirming functional redundancy in the GAPARAP subfamily^30^. The observation that rescue of neurotoxicity, can require at least two points of simultaneous modulation at highly pleiotropic targets, even in an acute *in vitro* model, highlights the need for targeted multiple interventions in complex diseases as has been demonstrated for the efficacy of oncology therapeutics^35^,^37^,^38^.

MPP^+^ treatment resulted in reduced protein degradation which was not reversed following the combined overexpression. The overexpressed proteins formed prominent autophagosome-like structures that co-stained with endogenous LC3B, although not with LAMP1, indicating that the network targets formed autophagosomes which were unable to undergo fusion with lysosomes. A possible explanation for this lack of fusion may be the reduction in lysosomal acididty which is observed rapidly after MPP^+^ treatment and may persist due to decreased cellular ATP levels.

This is, to our knowledge, the first time that such a network approach has been used to identify proteins whose overexpression results in decreased toxin vulnerability in a neurological disease model. These data demonstrate that construction and analysis of protein-protein interaction networks can identify proteins that, when modulated in concert, demonstrate a synergistic ‘network’ effect in a PD model. Our observations support many of the tenets of network pharmacology and suggest that this approach may offer a paradigm for the identification of intervention points in complex diseases such as PD.

## Methods

### Construction of networks

A putative list of PD associated proteins was constructed using PD related reviews, the PD KEGG pathway^41^ and the OMIM database. Each of these was further analysed using PubMed to ensure there was evidence of each protein in both PD pathology and MPP^+^/MPTP induced cell death. From this seed list two shorter lists describing mitochondrial dysfunction and autophagy were created, the proteins for inclusion were determined manually following a literature review (Table 1). iRefIndex^27^ was downloaded on 19th July 2012, the non-human interactions were removed and an ID conversion table was created using iRefR^39^.The resulting MITAB table was converted to an edge list and then a network. Shortest path sampling was used to identify the nodes in the set of shortest paths between all seeds, all edges between this list of nodes are then included. Using this method and the two seed lists, two sub-networks were created, one describing mitochondrial dysfunction and the other autophagy.

### Analysis of networks

Two networks were created using the igraph^40^ package in R, one modelling the role of mitochondrial dysfunction in MPP^+^ cytotoxicity and the other representing the role of autophagy. The union of the mitochondrial and autophagic networks was formed using Gephi and the BC of each node in both the individual networks and the union network calculated. The ∆BC was then calculated as the BC in the union network minus the BC in each of the sub-networks: 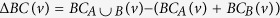. All networks were arranged using the Gephi force atlas algorithm, coloured according to original sub-network and nodes sized according to BC.

### Cell culture

The human dopaminergic neuroblastoma cell line BE(2)-M17 referred to as M17 was used throughout the investigation (ECCAC 95011816). Cells were cultured in Opti-MEM (OM) supplemented with 10% (v/v) foetal bovine serum, 100 U/ml penicillin and 0.1 mg/ml streptomycin at 37 °C and in 5% CO_2_. Prior to treatment, cells were seeded in 6 or 12-well plates at a seeding density of 1 × 10^5^ cells/mL; plates were pre-treated with poly-L-lysine. Cells were treated with MPP^+^ (20 nM - 2 mM) in antibiotic free media for periods from 1 to 48 h.

Knockdown of target proteins was achieved using siRNA in an RNase free environment. The following siRNAs (all Life Technologies were used at a 50 nM concentration: ScramSN1 (AM16106; GAGAAUAGGGAGGAGAACAtt), P62 (s16962; CUUCCGAAUCUACAUUAAAtt) and GABARAP (s22362; AGAAGAUCCGAAAGAAAUAtt); ScramSN1 was used as a control for the transfection process. Knockdown was conducted as previously reported^13^.

Cell transfection for overexpression used 1.25 μg plasmid DNA for each well of a 12-well plate with following plasmids: DsRed-P62 and HA-P62 gifts of Qing Zhong (Addgene plasmids #28024 and #28027), pEGFPc1-GABARAP, pEGFPc1-GBRL1 and pEGFPc1-GBRL2 all kind gifts of Kunikazu Tanji^29^.

### Western blot

After treatment, cells were washed once with unsupplemented OM and then lysed in RIPA buffer. Samples were separated by SDS-PAGE and transferred onto PVDF membranes. Membranes were blocked for 2 hours at room temperature in western blot buffer [Tris-buffered saline, 1% (v/v) Tween-20, 5% w/v skimmed milk] and then incubated with primary antibodies in western blot buffer overnight at 4 °C. The following primary antibodies were used: rabbit anti-LC3B (1:500, Sigma, L7543), rabbit anti β-Actin, anti-COX IV and anti-ubiquitin (1:2000; ab8227, 1:1000; ab16056 and 1:500; ab7780 respectively), mouse anti-P62 (1:2000, Abcam, ab56416), rabbit anti-GABARAP, anti-GBRL1 and anti-GBRL2 (1:1000, Source BioScience Lifesciences, 18723-1-AP, 11010-1-AP and 18724-1-AP respectively). Membranes were washed three times, (TBS 1% (v/v) Tween-20) then the appropriate horseradish peroxidase-conjugated secondary antibodies were applied for 1 h at room temperature, suspended in western blot buffer (1 in 5000 dilution). Blots were visualised using western chemiluminescent HRP substrate and images were captured using Chemidoc XRS. The intensity of each band was quantified using Gel Analysis tool in ImageJ, expression was normalised to actin expression levels and expressed relative to untreated cells.

### Microarrays

RNA was extracted from control and MPP^+^ treated cells (100 μM, 24 h, 6 well plates) prior to microarray analysis using the RNeasy Mini Kit (Qiagen). Extracted RNA was stored at −80 °C prior to microarray analysis. RNA Integrity Numbers (RIN) were calculated to assess the quality of the RNA. Where RIN values were satisfactory, expression analysis was performed using Affymetrix Human Gene 2.0 ST Arrays. The Probe Logarithmic Intensity ERror (PLIER) algorithm was used to calculate gene expression using a probe affinity parameter to correct for differing binding affinities. Expression values for each probe were calculated for each sample and average fold changes following MPP^+^ treatment were found. P-values were calculated using a multiple t-test and multiple testing corrections were performed.

### Assessment of cellular viability

Cells were cultured for 1–48 h following treatment with MPP^+^ in triplicate. Cells were viewed under bright-field microscopy (10 × magnification); three representative fields of view were photographed for each well. A 400 × 400 pixel region was randomly selected within each image and morphologically normal cells (defined as those with intact processes) were manually counted using ImageJ.

Other viability assays were conducted using a plate reader. All conditions were conducted in triplicate and average values for blank wells were subtracted to give a value for each treatment condition. Values were normalised to untreated cells to give a percentage viability for each treatment condition.

Cell proliferation was measured using Promega Cell Titre 96 AQueous Non-Radioactive Cell Proliferation Assay (MTS ((3-(4,5-dimethylthiazol-2-yl)-5-(3-carboxymethoxyphenyl)-2-(4-sulfophenyl)-2 H-tetrazolium) and electron coupling reagent phenazine methosulfate (PMS)). Cells were incubated for 2 h with the reagent and absorbance measured at 490 nm to assess the extent of MTS to formazan conversion.

Cellular ATP was measured using CellTiter-Glo Luminescent Cell Viability Assay (Promega) according to manufacturer’s instructions.

Tetramethylrhodamine (TMRM) fluorescence was used to assess mitochondrial membrane depolarisation. Cells were incubated with 150 nM TMRM for 5 min and then washed four times with PBS. Fluorescence was measured using a plate reader (excitation 544 nm, emission 590 nm).

Neutral red absorbance was used to measure cellular viability, as previously published^41^. Briefly, cells were treated with neutral red 40 μg/L for 2 h and then washed with PBS. Destain solution (500 μL, 50% (v/v) ethanol, 1% (v/v) glacial acetic acid) was then added to each well and plates were shaken for 5 min to ensure even mixing. Absorbance was measured at 540 nm.

### Assessment of autophagic protein degradation

Protocol for assessing autophagic protein degradation using radio-label pulse-chase assay were based on methods of Gronostajski and Pardee^42^. Cells were seeded in 6-well plates at a density of 1 × 10^5^ cells/mL and then incubated for 24 h with [^14^C]-valine (0.25 μCiml^−1^) to ensure labelling of long lived proteins. Cells were then washed and, where designated, transfections were performed as above. After an additional 12 h cells were washed again and media was replaced with [12 C]-valine enriched media (1 mM) to allow for the breakdown of short-lived proteins. A total of 24 h after the removal of the radio-label the cells were again washed and the media replaced (new media contained [^12^C]-valine (1 mM) and rapamycin (1 μM) or MPP^+^ (100 μM)) where indicated. Aliquots of media were taken at the timepoints indicated and precipitated with equal volumes of ice cold trichloroacetic acid (TCA). Tubes were then centrifuged for 20 min at 600 g and supernatant containing acid-soluble radioactivity counted using a liquid scintillation counter. At the final time point, cells were lysed with NaOH (0.1 M) and the radioactivity of the cellular fraction measured using a liquid scintillation counter. Proteolysis was defined as the ratio of labelled amino acids in the acid soluble fraction to the total amount in both the acid soluble and cellular fractions.

For immunocytochemistry, cells were grown on poly-L-lysine coated coverglasses. At the indicated time points cells were fixed for 15 min with 4% (w/v) paraformaldehyde and then maintained at 4 °C in PBS. Cells were blocked for 2 h at room temperature (PBS10% (v/v) normal goat serum and 0.1% (v/v) Triton-X100). Cells were incubated overnight (PBS 1% (v/v) normal goat serum and 0.1% (v/v) Triton-X100) with the following antibodies and concentrations: mouse anti-P62 (1:1000; Abcam; ab56416), rabbit anti-LC3B (1:100; Cell Signalling; 2775), rabbit anti-Tom20 (1:1000; Santa Cruz; sc-11415), rabbit anti-LAMP2 (1:500; Abcam; ab18528) and rabbit anti-ubiquitin (1:100; Abcam; ab7780).

Cells were washed and incubated for 1 h at room temperature with appropriate fluorescent antibodies (PBS 1% (v/v) normal goat serum and 0.1% (v/v) Triton-X100). Cells were washed and incubated for 10 min with DAPI (400 pg/mL). After further washing coverglasses were fixed to slides, stored at 4 °C protected from light. Cells were imaged using the EVOS FL Auto (60 × magnification) using filters for DAPI, GFP, Tx-Red and Cy5 – all images were merged in ImageJ.

### Statistical analysis

Data were normalised relative to untreated control cells and presented as a mean percentage of this value. Comparisons between two groups were made using a two-tailed t-test. Multiple comparisons were assessed using a one-way analysis of variance with a Dunnett’s post-hoc test for multiple comparisons. P-values <0.05 were considered statistically significant after correction for multiple testing.

## Additional Information

**How to cite this article**: Keane, H. *et al.* Protein-protein interaction networks identify targets which rescue the MPP^+^ cellular model of Parkinson’s disease. *Sci. Rep.* **5**, 17004; doi: 10.1038/srep17004 (2015).

## Supplementary Material

Supplementary Information
