# Supplementary material for: Protein-protein interaction networks identify targets which rescue the MPP+ cellular model of Parkinson’s disease
Source: Sci Rep. 2015 Nov 26;5:17004. doi: 10.1038/srep17004 (PMC4660280; doi:10.1038/srep17004)

Supplementary material:

Protein-protein interaction networks identify targets which rescue the MPP+ cellular model of Parkinson's disease

Harriet Keane†, Brent J. Ryan†, Brendan Jackson, Alan Whitmore and Richard Wade-Martins*

Supplementary Figure 1: Expression of network targets is unaltered following MPP^+ exposure.

Expression of networks targets was unaltered following MPP^+ exposure. (**a-c**) Protein levels of network targets was assessed by western blot following MPP^+ treatment (48 h, 100 µM) (**d**) RNA was harvested from control and MPP^+ treated cells and analysed using Human Gene 2.0 ST Arrays. Probe signal correlates with gene expression. mRNA expression of network targets was unaltered. (**e-g**) The effect of MPP^+ on protein levels of network targets was quantified by western blots with band intensities normalised relative to actin. Bars represent mean ± SEM (n=3).

Supplementary Figure 2: MPP^+ induces cell death in a time and dose dependent manner via mitochondrial membrane depolarisation.

Cellular viability following MPP^+ treatment. (**a**) Cell viability was assessed by the MTS assay following MPP^+ treatment. (**b** and **c**) Cell death was assessed by counting the number of morphologically normal cells in bright field images following MPP^+ treatment. (20 x magnification, scale bar is 100 µm). (**d**) MPP^+ (100 µM, 5h) increases lysosomal pH as assessed by neutral red accumulation. The vacuolar-type H^+-ATPase inhibitor bafilomycin A1 (50 nm, 5h) was used as a positive control for lysosomal deacidification. (**e**) Cell viability was assessed by neutral red in normal or low glucose media following MPP^+ treatment (100 µM). (**f**) Cellular ATP was also measured in normal or low glucose media following MPP^+

treatment (100 µM). In low glucose, high galactose media there is no net release of ATP from glycolysis and energy production is entirely dependent on oxidative phosphorylation as evidenced by potentiation of MPP^+ toxicity in low glucose conditions. (**g**) Mitochondrial membrane polarisation was measured using TMRM fluorescence following MPP^+ treatment (5 or 100 µM). Data points represent mean values normalised to untreated control cells ± SEM (n=3), *= P ≤0.05, ***=P ≤0.001 compared to untreated controls.

Supplementary Figure 3: Overexpression of GABARAP subfamily proteins.

Cells were transfected with GABARAP-EGFP subfamily fusion protein constructs. (**a**) Cells were imaged 72 h after transfection with either individual constructs or the combination of GABARAP, GBRL1 and GBRL2 (20 x magnification, scale bars are 70 µm). (**b**) Fluorescent cells were counted and the proportion of fluorescent cells was calculated. Bars represent mean ± SEM (n=3). (**c**) Extended blots (originally shown in *Figure 3b*) demonstrating both endogenous and GFP fusion forms of the GABARAP proteins 72 h after indicated transfection.

Supplementary Figure 4: MPP^+ causes autophagosome accumulation associated with failed degradation.

Autophagic flux following MPP^+ treatment measured using LC3B lipidation and pulse-chase assay. (**a** and **b**) Cells treated with MPP^+ and/or bafilomycin were harvested 48 h after treatment. LC3B levels were measured by western blot and the ratio of LC3B-II to LC3B-I calculated. Bars represent mean ± SEM (n=3), *= P ≤0.05. (**c**) The rate of protein degradation in 14C labelled cells was measured following treatment with MPP^+ (100 µM) or rapamycin (1 µM), linear regression was performed and lines plotted.

Supplementary Table 1: Mitochondrial seed proteins.

Proteins which have independently been experimentally shown to contribute to mitochondrial dysfunction in an MPP^+ model.

Supplementary Table 2: Autophagy seed proteins.

Proteins which have independently been experimentally shown to contribute to alterations in autophagy following MPP^+ treatment.

Tables

Supplementary Table 1

**Protein**	**Function**
Heat shock protein 75 kDa	Phosphorylated by PINK1, mitochondrial analogue of Hsp90, binds to TNF type 1 receptor associated protein [43].
Dynamin-1-like protein	A key mediator of mitochondrial fission [44].
Transcription factor A, mitochondrial	Mitochondrial transcription factor. Pro-survival. Decreases ROS production [45].
Bax	Proapoptotic, cytosolic protein which insets into membranes upon apoptotic stimuli. Regulated by p53 [46]
Parkin	Ubiquitin ligase. Removes abnormally folded proteins. Downstream of PINK1. Important for mitochondrial fission [47-49].
DJ-1	Removes reactive oxygen species. Involved of regulation of mitochondrial dynamics though DLP [50,51].
HTRA2	Mitochondrial serine protease, relocates to cytosol leading to initiation of apoptosis [52].
VDAC1	Voltage-gated anion channel, regulates ion transport between mitochondria and cytosol [53].
PINK1	A kinase important for the activation of parkin-mediated mitophagy, in addition to parkin-independent roles in mitochondrial dynamics [8,54]

Supplementary Table 2

**Protein**	**Function**
Microtubule-associated proteins 1A/1B light chain 3B (LC3b)	Lipidated upon the induction of autophagy and a key component of the autophagasome membrane [55].
LAMP2A	Lysosomal membrane glycoprotein, interacts with HSC70 to facilitate chaparone-mediataed autophagy (CMA) of substrates including α-syn. Post-translationally modified or mutant forms of α-syn impair CMA [56,57].
Hsc70-interacting protein	An adaptor protein which facilitates the transport of CMA subtrates through LAMP2a. Hsc70 recognises a pentapeptide sequence to on CMA substrates [58].
Ambra1	Activator of autophagy stabilized by autophagic stimuli by disrupting cullin-4 interaction [59]
Beclin-1	An Atg6 orthologue important for initiation of autophagy. Beclin-1 has several domains facilitating its interaction with pro-autophagy/pro-apotosis molecules indicating its role in determination of cell fate [60]
UCHL1	Ubiquitin carboxy-terminal hydrolase L1, an enzyme abundant in neurons which catalyses the hydrolysis of C-terminal ubiquitin adducts, facitlitating ubiquitin monomer formation and hence ubiquitin dynamics [61]
P35	Neuronal-specific CDK5 activator, regulation of protein activity occurs primarily through post-translational calpain cleavage [62]

Suplementary Figure 1

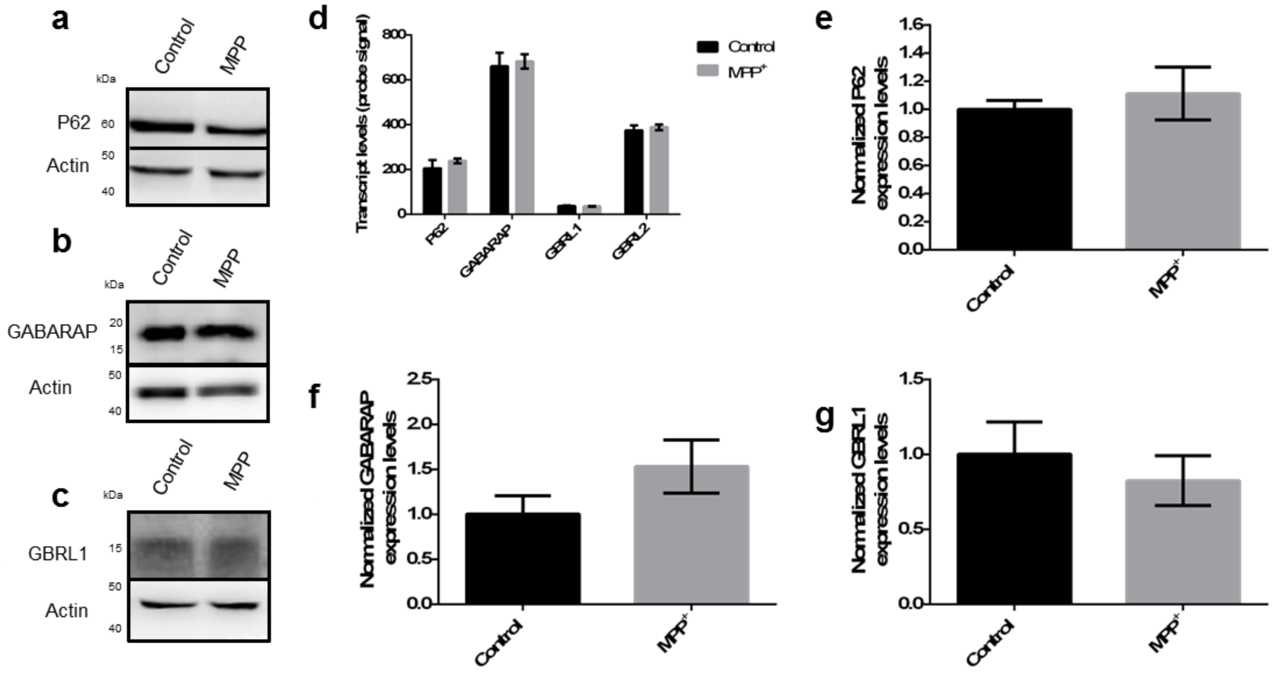

Suplementary Figure 2

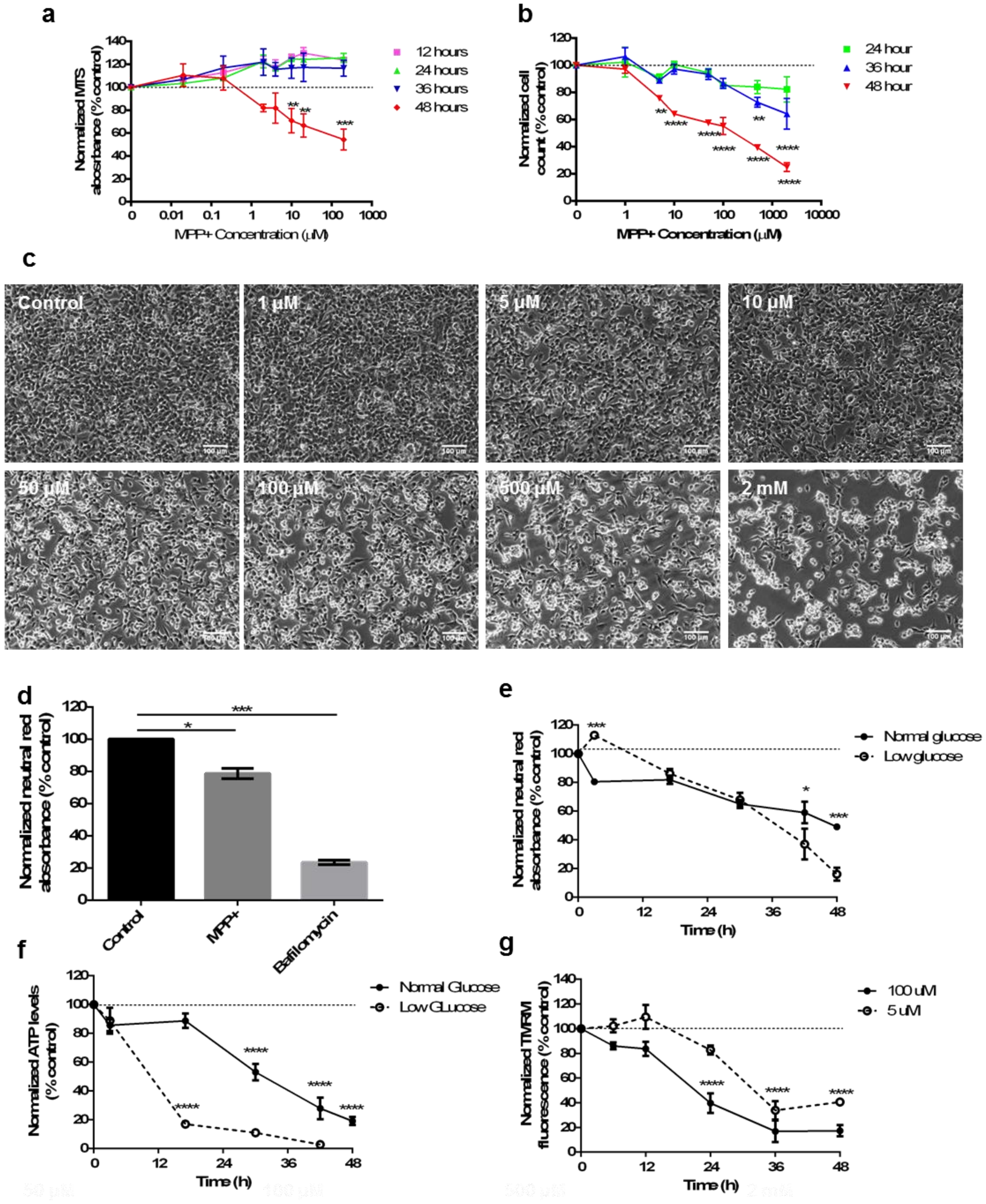

Suplementary Figure 3

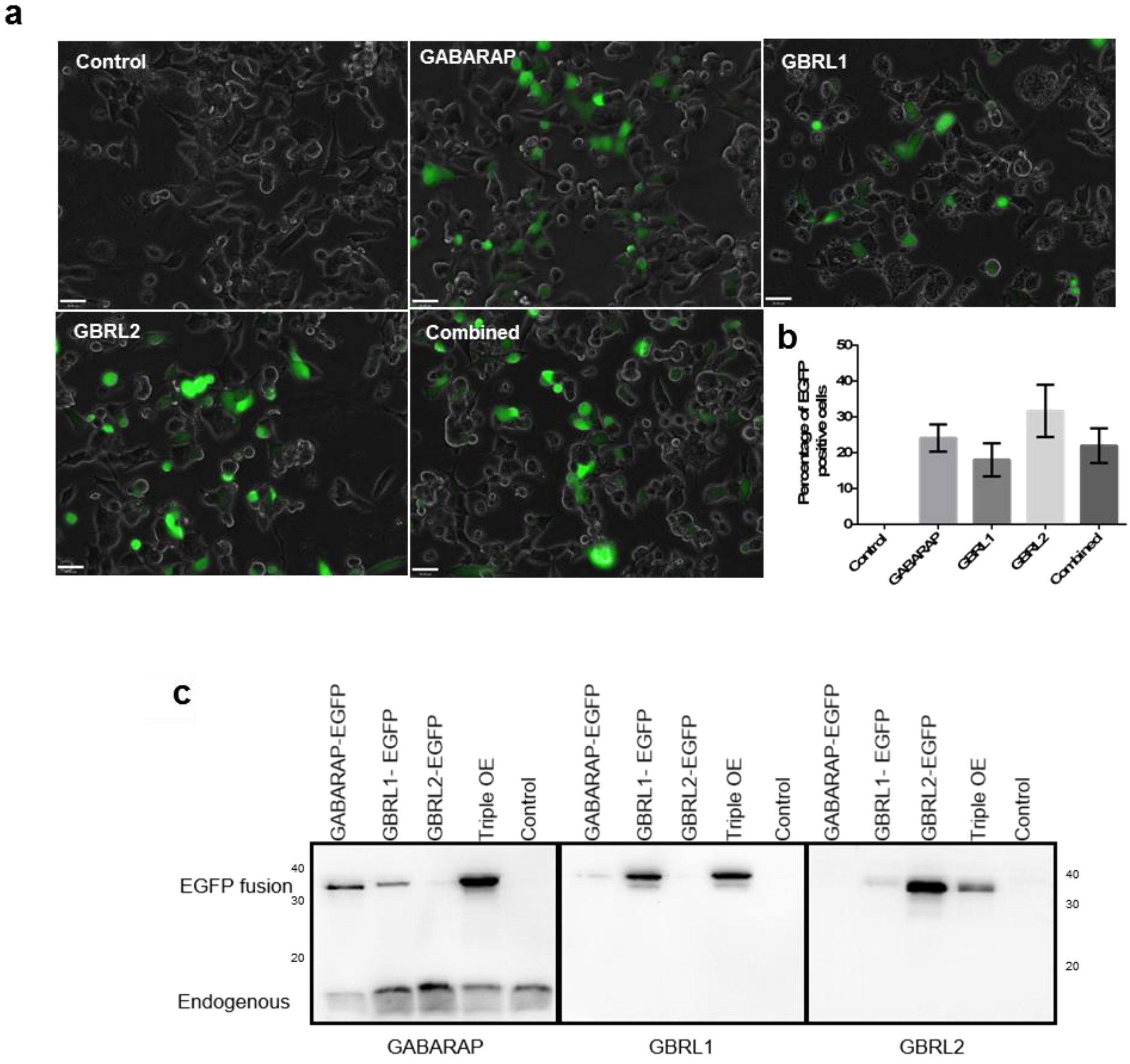

Suplementary Figure 4

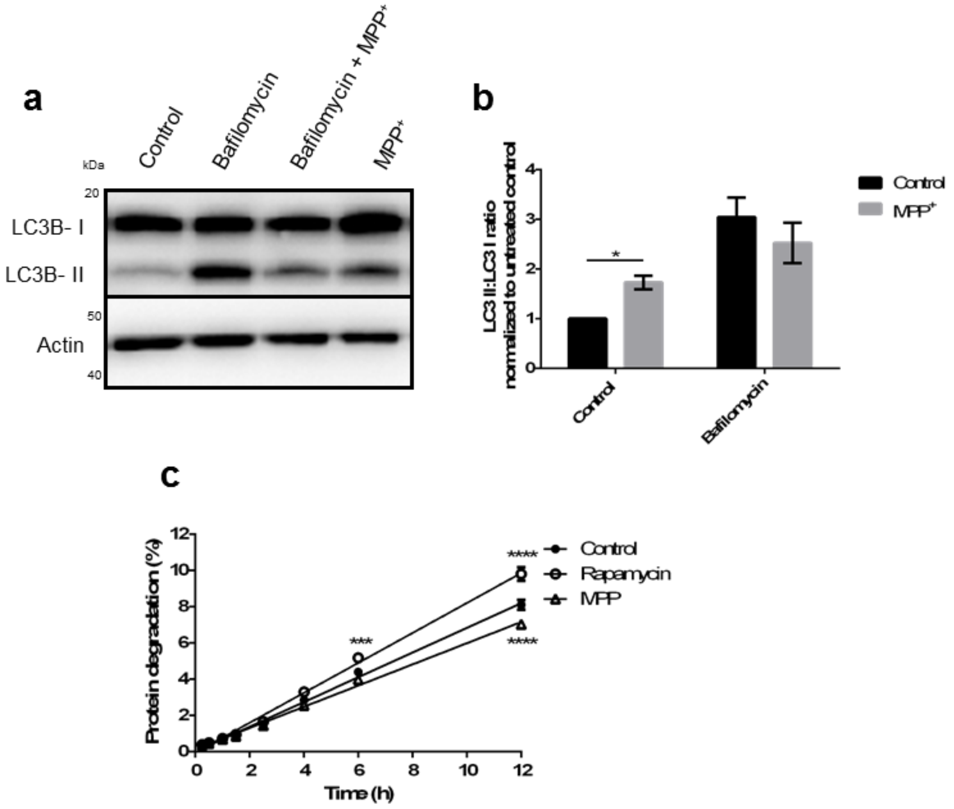

Supplement: Supplementary Information [file srep17004-s1.pdf]
